# Effect of Medium-Chain-Length Alkyl Silane Modified Nanocellulose in Poly(3-hydroxybutyrate) Nanocomposites

**DOI:** 10.3390/polym16213069

**Published:** 2024-10-31

**Authors:** Cătălina Diana Uşurelu, Denis Mihaela Panaitescu, Gabriela Mădălina Oprică, Cristian-Andi Nicolae, Augusta Raluca Gabor, Celina Maria Damian, Raluca Ianchiş, Mircea Teodorescu, Adriana Nicoleta Frone

**Affiliations:** 1 National Institute for Research & Development in Chemistry and Petrochemistry—ICECHIM, 202 Splaiul Independentei, 060021 Bucharest, Romania; catalina.usurelu@icechim.ro (C.D.U.); madalina.oprica@icechim.ro (G.M.O.); cristian.nicolae@icechim.ro (C.-A.N.); raluca.gabor@icechim.ro (A.R.G.); adriana.frone@icechim.ro (A.N.F.); 2 Faculty of Chemical Engineering and Biotechnology, National University of Science and Technology Politehnica, 011061 Bucharest, Romania; celina.damian@upb.ro (C.M.D.); mircea.teodorescu@upb.ro (M.T.)

**Keywords:** polyhydroxyalkanoates, cellulose nanofibers, alkyl silane, AFM, nanodimension

## Abstract

Poly (3-hydroxybutyrate) (PHB) is a valuable biopolymer that is produced in industrial quantity but is not widely used in applications due to some drawbacks. The addition of cellulose nanofibers (CNF) as a biofiller in PHB/CNF nanocomposites may improve PHB properties and enlarge its application field. In this work, n-octyltriethoxy silane (OTES), a medium-chain-length alkyl silane, was used to surface chemically modify the CNF (CNF_OTES) to enhance their hydrophobicity and improve their compatibility with PHB. The surface functionalization of CNF and nanodimension were emphasized by Fourier transform infrared spectroscopy, X-ray photoelectron spectroscopy, thermogravimetric analysis, atomic force microscopy, dynamic light scattering, and water contact angle (CA). Surface modification of CNF with OTES led to an increase in thermal stability by 25 °C and more than the doubling of CA. As a result of the higher surface hydrophobicity, the CNF_OTES were more homogeneously dispersed in PHB than unmodified CNF, leading to a PHB nanocomposite with better thermal and mechanical properties. Thus, an increase by 122% of the storage modulus at 25 °C, a slight increase in crystallinity, a better melting processability, and good thermal stability were obtained after reinforcing PHB with CNF_OTES, paving the way for increasing PHB applicability.

## 1. Introduction

Poly (3-hydroxybutyrate) (PHB) is an aliphatic polyester from the class of polyhydroxyalkanoates (PHA). It is biodegradable and biocompatible and is industrially obtained through bacterial synthesis [1,2]. PHB can be produced by a great number of bacterial strains, but currently *Cupriavidus necator* is the most used on an industrial scale due to its large PHB production using cheap substrates derived from wastes [1]. PHB stands out for its high crystallinity and good gas barrier properties, which, together with its high percent biodegradation in different environments (marine, soil, industrial, or home composting), make it a great sustainable material for single-use food packaging [1,2]. PHB is also a promising material for biomedical applications because it is biocompatible, not toxic, and its degradation products are also not toxic [1,2].

Although PHB is available on the market and has been intensively studied in recent years, it is not widely used in applications due to some deficiencies, the most important of which are high brittleness, difficult melt processing due to the small temperatures’ range that allows processing without degradation, and a low melt viscosity and strength [2]. One of the most accessible ways to improve the properties of biopolymers and widen their applicability is to reinforce them with cellulose nanofibers (CNFs), which are obtained from cellulose, a biodegradable and widespread polymer in nature, by mechanical treatments [3,4]. CNFs stand out by a high surface area, an elevated aspect ratio, and very good mechanical properties; however, the abundance of the -OH groups on their surface, which confers a high reactivity for a multitude of chemical modifications, also gives great hydrophilicity, which is a shortcoming when CNFs are used as reinforcing fibers in hydrophobic polymers [5]. Therefore, the chemical surface modification of CNFs is mandatory for a good dispersion of CNFs in hydrophobic polymers [6].

Several attempts have been made to chemically modify the surface of nanocellulose for increased compatibility with the PHB matrix, but most of them were performed on cellulose nanocrystals (CNCs) [4]. Thus, CNCs grafted with poly(3-hydroxybutyrate-co-3-hydroxyvalerate) (PHBV) and CNCs grafted with polylactide were used as reinforcing agents in PHBV [7] and PHB [8] nanocomposites, respectively, while CNCs surface modified with butyric acid, lactic acid, and their mixture were employed to reinforce PHBV [9]. The chemical modification of CNFs by esterification, oxidation, amidation, and silylation to increase their interfacial compatibility with another aliphatic polyester matrix, polylactic acid (PLA), is well documented in many studies [10,11], but the chemical modification of CNFs for serving as reinforcing fibers in nanocomposites based on PHB or other polyhydroxyalkanoates is very scarce. Popa et al. [12] tried to improve the compatibility between PHB and CNFs by the graft polymerization of methacrylic acid on the CNF surface previously activated with γ-methacryloxypropyltrimethoxy silane. As a result of this modification, the nanocomposites showed uniform dispersion of the surface-treated CNFs in the polymer matrix, smaller PHB spherulites, good thermal stability, and mechanical properties improved by up to 23% [12]. In another attempt, TEMPO oxidation was used for the defibrillation and surface functionalization of microcrystalline cellulose, which was further used as a reinforcing agent in PHB [13]. The PHB composite containing TEMPO-modified cellulose presented better mechanical properties, with a 20% increase in storage modulus and an improved fiber–polymer interface than the composite with untreated cellulose fibers [13]. The treatment of CNFs with low molecular weight styrene/maleic anhydride copolymers (SMA) was also reported [14]. Although the treatment conditions, i.e., mixing the components in water at room temperature for 24 h without any catalyst, probably did not ensure the covalent bonding of the SMA to the CNFs surface, a 17% increase in the maximum tensile strength and a 24.5% increase in modulus were noticed for PHB/SMA-coated CNFs compared to the composite containing untreated fibers [14]. Further, strong interactions were observed between PHB and defibrillated fibers obtained from wood waste in melt-compounded composites due to the presence of lignin with hydrophobic groups on the surface of the cellulose fibers [15]. These interactions led to a significant improvement in the thermal and mechanical properties of the PHB composite, with a 22% increase in the storage modulus being reported for the composite as compared to neat PHB. Therefore, the use of CNF reinforcements chemically modified on their surface with compounds containing hydrophobic groups could be a good solution to amend PHB’s deficiencies.

Organosilanes are a class of modifiers that can impart new properties to the CNFs, such as increased hydrophobicity, improved humidity resistance, and increased compatibility with biopolymer matrices [11,16]. Alkyl silanes with different lengths of the alkyl chains were tested as modifiers for cellulosic substrates [16,17]. Olive husk flour, which was surface modified with trimethoxyoctadecyl silane using a thermochemical vapor deposition at 120 °C for 48 h and further added as a filler (20 wt%) in PHBV, determined a higher reinforcing effect than the untreated flour [16]. In this case, the long-chain alkyl silane-treated CNFs caused an increase in the water contact angle, crystallinity, storage, and Young’s moduli [16]. In more recent work, Ou et al. studied the influence of two n-alkyl silanes, methyltrimethoxy silane (MTMS), having a very short chain, and hexadecyltrimethoxy silane (HDTMS), with a long aliphatic chain, on the hydrophobic properties of a cotton fabric, to which they were chemically anchored [17]. While the MTMS treatment did not modify the hydrophilicity of the cellulose substrate, the HDTMS treatment rendered the cotton fabric hydrophobic (water contact angle around 155°) [17]. Similarly, microfibrillated cellulose (MFC) of about 10 μm in width, obtained starting from oat hull fiber, was surface treated with methyltriethoxy silane in the presence of ammonium hydroxide and employed to prepare poly(3-hydroxybutyrate-co-3-hydroxyhexanoate)/10 wt% MFC composites by melt compounding using a corotating twin-screw extruder [18]. The composite with silane-treated MFC showed similar Young’s modulus and lower tensile strength than the composite obtained with untreated fibers in the same concentration. Therefore, short-chain-length alkyl silanes treatment of CNFs does not lead to satisfactory properties in PHA/CNF nanocomposites, and the length of the aliphatic chain in n-alkyl silanes is an important characteristic of the surface-modified CNFs because it determines the efficiency of the alkyl silanes in imparting hydrophobic properties to the cellulose substrate. In a similar study conducted on mesoporous silica particles, which were modified with n-alkyl silanes with C3, C8, C12, and C18 alkyl chains, Pyo and Chang observed an increase in the contact angle with the increase in the length of the alkyl chain of silanes [19]. The extent of the formation of the Si-O-Si cross-linked structures on the surface of mesoporous silica particles was analyzed using solid-state nuclear magnetic resonance, and the results showed a decrease in the Si (T3) integrations with the increase in the alkyl chain length, which is a steric hindrance effect [19]. Therefore, a long-chain alkyl silane modifier could impede the self-condensation and formation of a cross-linked siloxane layer on the cellulose substrate, which is a desired effect for surface hydrophobized CNFs. However, the hydrolysis reaction of long-alkyl-chain silanes in protic solvents and their grafting on cellulosic substrates are very slow processes, which leads to a small efficiency of the processes [19]. Moreover, previous studies have shown that only the treatment with alkyl silanes having more than seven carbon atoms in the alkyl chain leads to a hydrophobic modification of a cellulosic substrate, with shorter alkyl chain silanes having small or no hydrophobization effect [17]. Therefore, a medium-chain-length alkyl silane such as n-octyltriethoxy silane (OTES) could be an efficient silylating agent for CNFs.

Previously, OTES was successfully grafted on cellulose beads to obtain superhydrophobic beads for oil-water separation [20]. The functionalization of the cellulose beads with OTES changed their surface from a highly hydrophilic to a superhydrophobic one, capable of separating oil/water mixtures even in harsh conditions caused by corrosive liquids [20]. Moreover, cellulose fibers silylated with OTES were used as a reinforcing agent in poly(methyl methacrylate) for dental applications, leading to a dental composite with better thermal and mechanical properties [21]. In another attempt, ground horsetail biomass was modified with OTES to improve its compatibility with natural rubber, a non-polar elastomer matrix [22]. As a result of the chemical functionalization of the lignocellulosic filler, its thermal stability, expressed by the temperature of 10% weight loss, was improved by about 60 °C, and the contact angle increased from 78.5° for the untreated filler to 114° for the OTES-treated filler [22]. The improved hydrophobic character of the horsetail fibers after OTES functionalization led to better homogeneity and mechanical properties of the natural rubber biocomposite obtained with this filler [22].

Considering the results obtained with other fillers and polymer matrices after the OTES treatment, in this work, OTES was used to surface chemically modify CNFs to enhance their hydrophobicity and improve their compatibility with a PHB matrix. Although CNFs were previously functionalized with other organosilanes [12,23] to enhance their compatibility with PHAs, this is the first study regarding the usage of a medium-chain-length alkyl silane to modify CNFs and to investigate their effect on the properties of PHB/CNF nanocomposites. The surface functionalization of CNFs and nanodimension were emphasized by Fourier transform infrared spectroscopy (FTIR), thermogravimetric analysis (TGA), atomic force microscopy (AFM), dynamic light scattering (DLS), and water contact angle, while their influence on the properties of PHB nanocomposites was monitored by differential scanning calorimetry (DSC), dynamic mechanical analysis (DMA), TGA, and scanning electron microscopy (SEM). The results showed that OTES-grafted CNFs are promising modifiers for PHB and may increase their applicability in single-use packaging.

## 2. Materials and Methods

### 2.1. Materials

Microcrystalline cellulose (MCC) with a mean particle diameter of 20 μm was purchased from Sigma-Aldrich (Saint Louis, MO, USA) and used as a source of CNF, while n-octyltriethoxy silane (OTES) (purity 97%, density 0.88 g/cm^3^) was provided by ThermoScientific (Waltham, MA, USA). Absolute ethanol (purity 99.3%, density 0.789 g/cm^3^) was acquired from PAM Corporation (Piteşti, Romania), and glacial acetic acid (purity 99.84%, density 1.05 g/cm^3^) was supplied by Chimreactiv (Bucharest, Romania). Poly(3-hydroxybutyrate) (PHB) pellets, commercial grade P304 with a density of 1.2 g/cm^3^, were sourced from Biomer (Schwalbach am Taunus, Germany). All commercial reagents and solvents were used as received without additional purification.

### 2.2. Isolation of CNF

CNFs were obtained by the mechanical defibrillation of a 1.5 wt% aqueous suspension of MCC in a microfluidizer LM20 (Microfluidics, Westwood, MA, USA) equipped with a Z-type diamond interaction chamber for 15 consecutive cycles at 150 MPa.

### 2.3. Chemical Modification of CNF with OTES

**Figure 1:**
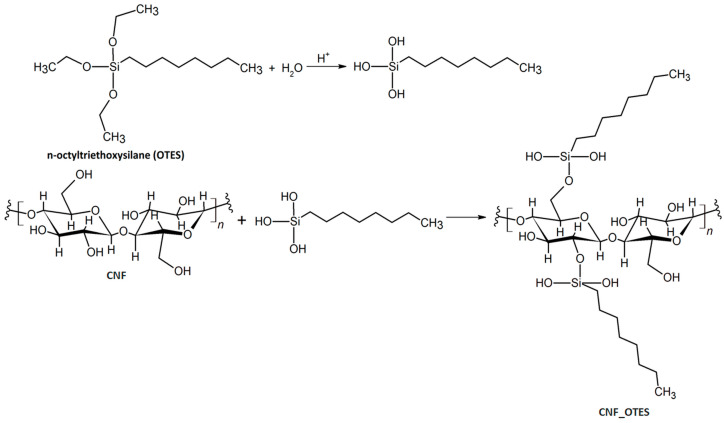
Chemical functionalization of CNF with OTES.

The grafting reaction of OTES on CNF involved the preparation of a 5 wt% OTES solution by the dissolution in a mixture of absolute ethanol and water (V_ethanol_/V_water_ = 95/5) of an amount of OTES corresponding to an OTES:anhydroglucose unit (AGU) molar ratio of 1:1, adjusting the pH of the mixture to a pH of 4.0–4.5 with acetic acid and allowing it to react at room temperature, under intense magnetic stirring, for 90 min, period necessary for the hydrolysis of the silane. Meanwhile, 1.5 wt% CNF aqueous suspension was centrifuged at 9000 rpm for 2 min at 20 °C to isolate it from water. The CNF sediment was then added to the hydrolyzed silane solution and allowed to react at room temperature under strong magnetic stirring for 3 h. Subsequently, the reaction mixture was transferred to a round-bottomed reaction flask coupled to a reflux condenser, placed in an oil bath heated to 100 °C, and allowed to further react under reflux and magnetic stirring for 1 h to complete the chemical grafting. Later, 52 mL of water was added to the reaction mixture, and the flask was transferred to an oven heated at 110 °C and maintained there for 30 min to perfect the silylation reaction. At the end of the reaction, the OTES-treated CNF (CNF_OTES) was isolated from the reaction mixture by centrifugation at 8000 rpm for 10 min at 20 °C and then subjected to Soxhlet extraction in 400 mL acetone for 8 h to remove unreacted OTES and/or oligosilanols that were not covalently grafted on the CNF surface. Afterward, the purified CNF_OTES was washed with water, separated by centrifugation at 8000 rpm for 15 min at 20 °C, then redispersed in water, and together with the untreated CNF, they were stored in a freezer for 24 h and then dried in a FreeZone 2.5 L Benchtop Freeze Dry System lyophilizer (Labconco, Kansas City, USA) at 0.006 Torr and −84 °C for 72 h. The hydrolysis of the silane and the reaction of silanol groups with CNF to give CNF_OTES are shown in Figure 1.

### 2.4. Characterization of the CNF_OTES

#### 2.4.1. Fourier Transform Infrared Spectroscopy (FTIR)

FTIR analysis was carried out on the freeze-dried CNF and CNF_OTES samples in the attenuated total reflectance (ATR) mode using a TENSOR 37 spectrometer (Bruker, Billerica, MA, USA). For each sample, the FTIR spectrum was recorded in the wavenumber domain from 4000 to 400 cm^−1^, with a resolution of 4 cm^−1^, by the accumulation of 16 scans.

#### 2.4.2. X-Ray Photoelectron Spectroscopy (XPS)

The surface chemical composition of freeze-dried CNFs before and after OTES grafting was determined by using an ESCALAB™ XI+ spectrometer from Thermo Scientific (Waltham, MA, USA) provided with a monochromated Al Kα excitation source (1486.6 eV) working in ultrahigh vacuum (2 × 10^−9^ mbar). XPS survey spectra were collected at a passing energy of 200 eV and an energy step size of 1 eV by the accumulation of 5 scans. The degree of surface substitution (DS) of the silylated CNFs was calculated based on the data extracted from the XPS survey spectrum of CNF_OTES after the following equation [24,25]:

(1) $$DS=\frac{M_{AGU}\cdot x}{{100\cdot M}_{Si}-M_{silane\;graft}\cdot x}$$

where M_AGU_ is the molecular weight of the AGU in cellulose (162.14 g/mol), x is the mass concentration of Si on the CNF_OTES’s surface calculated using the Si 2p atomic concentration obtained from the XPS survey spectra, M_Si_ is the molecular weight of Si (28 g/mol), and M_silane graft_ is the molecular weight of the OTES graft (191 g/mol).

#### 2.4.3. Static Contact Angle (CA)

The contact angle measurements were performed using a CAM 200 optical contact angle meter (Biolin Scientific, Espoo, Finland) provided with a high-resolution camera (Basler A602f) and an auto-dispenser at room temperature, using deionized water. For this, aqueous suspensions of CNF and CNF_OTES, 1.5 wt% in concentration, were cast on glass slides and allowed to dry at room temperature. The CA value was assessed 1–2 s after the water drop touched the material’s surface. Five drops of water were placed at five different locations on each sample, and the reported CA represents the average of these five determinations. The CA values were calculated using the CAM 200 software.

#### 2.4.4. Thermogravimetric Analysis (TGA)

The thermal analysis of CNF and CNF_OTES was performed on freeze-dried samples sealed in platinum pans using a TGA Q5000 analyzer (TA Instruments Inc., New Castle, DE, USA). The tests were carried out from room temperature to 700 °C at a steady heating rate of 10 °C/min, with nitrogen as the purge gas flowing at 40 mL/min.

#### 2.4.5. Atomic Force Microscopy

The AFM instrument used in this analysis was a Multimode 8 microscope from Bruker (Santa Barbara, CA, USA) endowed with a Nanoscope V controller and PeakForce (PF) Quantitative Nanomechanical Mapping (QNM) module. The measurements were conducted in the air at room temperature using silicon probes with a nominal tip radius of 8 nm, a spring constant of 40 N/m, and a resonant frequency of 300 kHz. The images were acquired with a digital resolution of 256 px × 256 px at a scanning rate of 1.0–1.2 Hz and further processed using NanoScope software version 1.20. For AFM samples, diluted aqueous suspensions of CNF and CNF_OTES (0.01 wt%) were ultrasonicated in an ultrasound bath for 5 min, then immediately cast onto glass plates measuring approximately 10 mm × 10 mm and allowed to dry overnight at ambient conditions. The size of the nanofibers was analyzed using free ImageJ software Version 1.8.0 developed by Wayne Rasband from the National Institutes of Health (Bethesda, MD, USA).

#### 2.4.6. Dynamic Light Scattering (DLS)

The average hydrodynamic diameters and size distributions of the CNF and CNF_OTES samples in aqueous dispersion were determined by DLS, using a Zetasizer Nano ZS ZEN 3600 instrument (Malvern Instruments, Worcestershire, UK) equipped with a 532 nm He-Ne laser and a scattering angle of 173°, at 25 °C. The measurements were conducted on diluted aqueous suspensions (0.01 wt%) that were previously ultrasonicated using an ultrasound bath for 5 min, and the data presented are the average values from 5 measurements for each sample. The zeta potential (ζ) was measured using the same instrument and concentration of the samples through the electrophoretic mobility technique or Laser Doppler Velocimetry. Distilled water, which, at 25 °C, possesses a viscosity of 0.8872 cP, a refraction index of 1.330, and a dielectric constant of 78.5, was used as a reference dispersing medium.

### 2.5. Preparation of the PHB/CNF and PHB/CNF_OTES Nanocomposites

The PHB/CNF and PHB/CNF_OTES nanocomposites were obtained by melt mixing a formulation containing 96 wt% PHB and 4 wt% CNF or CNF_OTES in the 30 cm^3^ mixing chamber of a Brabender LabStation (Brabender GmbH & Co. KG, Duisburg, Germany) at 160 °C, a rotor speed of 40 rpm, for 6 min. The resulting compounds were subsequently passed on a laboratory two-roll mill (Polymix 110L, Duisburg, Germany) to shape them into sheets and then compression molded into films (0.60–0.65 mm in thickness) on an electrically heated press P200E (Dr. Collin, Maitenbeth, Germany) at 170 °C under the following conditions: pre-heating at 0.5 MPa for 120 s, compression at 10 MPa for 60 s, and cooling with a cooling cassette at 0.5 MPa for 60 s. A PHB reference was obtained in the same conditions by melt mixing, shaping on the laboratory two-roll mill, and compression molding.

### 2.6. Characterization of the PHB/CNF and PHB/CNF_OTES Nanocomposites

#### 2.6.1. Mixing Torque

The modifications in the melt viscosity of PHB following the addition of CNF or CNF_OTES were evaluated using the torque vs. time data recorded directly by the Brabender LabStation mixer and retrieved using the WINMIX software, version 3.2.30.

#### 2.6.2. Thermogravimetric Analysis of Nanocomposites

The TGA results for neat PHB, PHB/CNF, and PHB/CNF_OTES nanocomposites were obtained using a TA-Q5000 device (TA instruments, New Castle, DE, USA) on samples weighing 20–27 mg contained in platinum crucibles, which were heated from room temperature to 700 °C at a heating rate of 10 °C/min under nitrogen flow (40 mL/min).

#### 2.6.3. DSC Analysis

The thermal behavior of the neat PHB, PHB/CNF, and PHB/CNF_OTES nanocomposites was examined using DSC Q2000 equipment (TA instruments, New Castle, DE, USA) on samples weighing 13–15 mg, under a helium flow of 25 mL/min, and at a heating/cooling rate of 10 °C/min. The applied protocol consisted of cooling the sample from room temperature to −85 °C and 3 min equilibration at this temperature, heating at 215 °C (1st heating cycle) and 2 min equilibration, cooling again to −85 °C (cooling cycle) and 2 min equilibration, and then heating again at 215 °C (2nd heating cycle). The degree of crystallinity (Xc) of the composites was calculated from the second heating cycle using the following equation:

(2) $$X_{c}(\%)=\frac{{∆H}_{m}}{{∆H}_{0}\times w_{PHB}}\times 100$$

where ΔH is the melting enthalpy of the composite (J/g), ΔH_0_ is the melting enthalpy of hypothetical 100% crystalline PHB (146 J/g [13]), and w_PHB_ is the weight fraction of PHB in the composite.

#### 2.6.4. SEM Analysis of Nanocomposites

The morphological features of the PHB/CNF and PHB/CNF_OTES nanocomposite films, such as the distribution of the untreated and treated fibers in the PHB matrix, were analyzed using a tabletop scanning electron microscope Hitachi TM4000 plus II (Hitachi, Tokyo, Japan), operated at an accelerating voltage of 10 kV and a working distance of around 9 mm. For this, the PHB and nanocomposite films were previously cryo-fractured in liquid nitrogen and sputter-coated with a 5 nm layer of gold.

#### 2.6.5. Dynamic Mechanical Analysis

The variation in the storage modulus with temperature was recorded for neat PHB and the prepared nanocomposites using a DMA Q800 (TA Instruments, New Castle, DE, USA) instrument on rectangular specimens with the length × width × thickness of 12.8 mm × 6.5 mm × 0.65 mm, in the multi-frequency strain mode (tension clamp), from −65 °C to 125 °C and at a constant heating rate of 3 °C/min and a load frequency of 1 Hz.

## 3. Results and Discussion

### 3.1. OTES-Modified CNF

#### 3.1.1. FTIR Analysis of CNF and CNF_OTES

**Figure 2:**
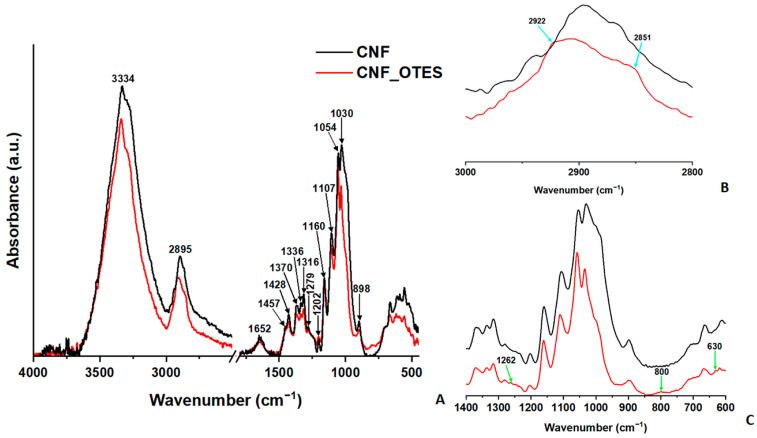
FTIR spectra of CNF and CNF_OTES (**A**); 3000–2800 cm^−1^ (**B**); and 1400–600 cm^−1^ regions (**C**).

Figure 2 shows the FTIR spectra of CNF and CNF_OTES. Pristine cellulose nanofibers present all the absorption bands characteristic of plant-derived cellulose, with a broad spectral band situated between 3700 and 3000 cm^−1^ attributed to the stretching vibrations of the hydrogen-bonded OH groups [26], another band between 2990 and 2800 cm^−1^ corresponding to the C−H stretching vibrations [26] in the methylene (CH_2_) and methine (CH) groups [27], and a minor absorption peak at around 1652 cm^−1^ ascribed to the O–H bending vibrations of absorbed water molecules [28]. Other absorption peaks were observed at around 1456 and 1428 cm^−1^ assigned to CH_2_ symmetric bending [29] and CH_2_ scissoring [30], 1370, 1336, and 1316 cm^−1^ assigned to CH bending [30], C-OH in-plane bending and CH_2_ wagging [31] and at 1279, 1248, 1235 and 1202 cm^−1^, respectively, ascribed to C–H in-plane bending [32], C−O−C stretching [33], and C–OH bending at the C6 carbon atom [31]. Other important bands in CNF, centered at around 1160, 1107, 1054, and 1030 cm^−1^ are associated with the C−O−C asymmetric stretching of the β-glycosidic linkages [27], skeletal C–OH stretching, C–O–C stretching of the pyranose ring [34], and C–O stretching [27]. Another peak specific to cellulose, situated at around 898 cm^−1^, describes the C–O–C stretching vibrations of the β-(1→4)-glycosidic linkages in CNF [35].

Several changes were observed in the FTIR spectrum of CNF after silylation with OTES (Figure 2). Specifically, the band between 3700 and 3000 cm^−1^, associated with hydrogen-bonded OH groups, narrowed, indicating a decrease in the number of OH groups available for hydrogen bonding interactions due to their partial conversion into O−Si−C following the silylation reaction or diminished water absorption [36] due to the surface modification of CNF with the hydrophobic OTES. The shift of the center of this band from 3334 to 3340 cm^−1^ also hints at a reduction in the hydrogen bonding interactions between the OH groups in CNF and/or a change in their nature by their conversion into O−Si−C groups following the coupling with OTES [37]. A shift of the center of another characteristic band, the one related to the CH symmetric stretching vibrations, from 2895 cm^−1^ to 2907 cm^−1^ was also noticed following the silylation with OTES. This could result from the spectral contribution of the C−H stretching vibrations of the methyl (CH_3_) and methylene (CH_2_) groups from the OTES grafts and from a decrease in the original hydrogen bonding in CNF [38]. Additionally, two new shoulders could be observed in the FTIR spectrum of CNF_OTES at around 2922 cm^−1^ and 2851 cm^−1^, which could be assigned to the C−H stretching vibrations of the CH_3_ and CH_2_ groups [39] from the aliphatic octyl chain of the OTES grafts.

In the 1190–920 cm^−1^ region, the peaks at 1160, 1107, 1054, and 1030 cm^−1^, associated with C–O stretching vibrations in the pristine CNF, likely overlap with the bands related to the Si−O−C and Si−O−Si stretching vibrations of the silane grafts, typically observed in the 1100–1010 cm^−1^ range [20]. Consequently, this region, otherwise extremely important, can offer limited evidence for the grafting of OTES onto CNF. However, similarly to the band from 3700 to 3000 cm^−1^, a narrowing of the 1043–920 cm^−1^ band, typically associated with the C−O stretching vibrations of the C–OH groups in CNF, was observed in the FTIR spectrum of CNF_OTES comparative with CNF, indicating that some of the OH groups of CNF were consumed in the silylation reaction with OTES. Stronger evidence of the covalent binding of OTES to CNF may be the new small peak that appeared at around 1262 cm^−1^ in the FTIR spectra of CNF_OTES that can be ascribed to stretching vibrations of the Si−CH_2_ groups in the OTES grafts [40]. The peak observed at around 800 cm^−1^, related to Si−O−C or Si−O−Si stretching vibrations [41], might also provide evidence for the covalent bonding of OTES to CNF, as well as the potential condensation between the silanol grafts anchored on the CNF’s surface. The appearance in the FTIR spectrum of CNF_OTES of a new absorption peak near 630 cm^−1^, assigned to the Si–O stretching coupled with O–Si–O and Si–O–Si bending [42], provides further proof that the chemical modification of CNF with OTES took place successfully.

Since the IR absorption bands characteristic of Si−O−C and Si−O−Si reside around 1100–1000 cm^−1^ [43], where they overlap with the intense stretching vibration bands of the C−O−C/C−O bonds in native cellulose, FTIR analysis may not be so conclusive to assess whether the chemical modification of CNF with OTES has occurred successfully. In addition, as shown by Kaynak et al. [44], OTES, the modifying agent, does not possess in its structure functional groups that can be readily distinguished from those of CNF in the FTIR spectra. Therefore, XPS spectra were recorded for both CHF samples.

#### 3.1.2. XPS Analysis of CNF and CNF_OTES

**Figure 3:**
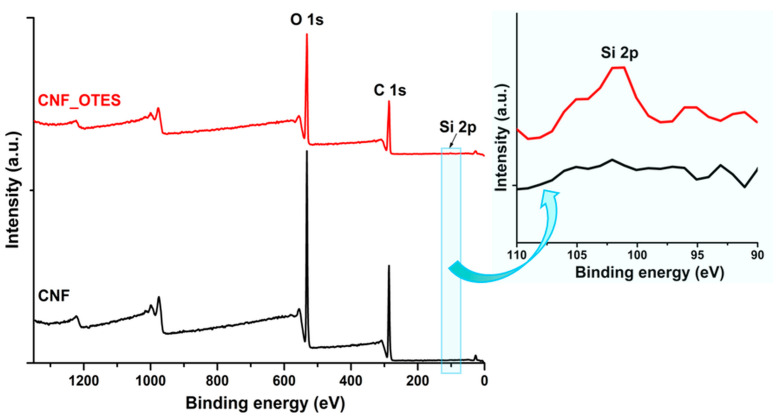
XPS survey spectra of CNF and CNF_OTES.

XPS is a powerful tool for assessing the extent of CNF silylation. It can offer semiquantitative information regarding the elemental composition at the surface of samples [45] and the changes in the elements’ bonding environment following the chemical modification reaction [46]. Figure 3 shows the XPS survey spectra of untreated CNF and silylated CNF, while Table 1 lists the relative atomic percentages and the O/C ratio at the neat CNF and CNF_OTES’ surfaces as computed from the XPS survey spectra.

The survey spectrum of CNF showed characteristic peaks at around 532 eV and 286 eV attributed to the oxygen (O 1s) and carbon (C 1s) atoms and no peaks related to silicon (Si), which is expected for unmodified CNF [42]. For CNF_OTES, two additional peaks were observed at ~152 eV and 102 eV corresponding to Si 2s and Si 2p, which testifies to the presence of silicon and, implicitly, of OTES grafts on the CNF_OTES’ surface [47]. The atomic concentration of Si 2p from the CNF_OTES sample was further used to assess the DS of CNF_OTES according to Equation (1). Thus, a relatively low DS of ~0.09 was determined for CNF_OTES. This is to be expected given the low OTES:AGU molar ratio of 1:1 (practically, no excess OTES) introduced in the silylation reaction. A lower DS of 0.243 mmol/g (corresponding to 0.04 mol/mol AGU) was reported by Coelho Braga de Carvalho et al. [48] for the silylation of CNFs with 3-mercaptopropyltrimethoxy silane in an ethanol/water mixture at RT for 3 h, followed by curing at 85 °C for 4 h. A slightly higher DS of 4.4% (corresponding to 0.13 mol/mol AGU) was obtained by Xu et al. [49] for the chemical treatment of CNFs with 3-(trimethoxysilyl)propyl methacrylate in an ethanol/water mixture at 70 °C for 48 h.

The O/C atomic ratio in pristine CNF, calculated from the XPS data, is 0.74 (Table 1). This is lower than the theoretical ratio of 0.83 computed based on the chemical formula of cellulose, probably due to surface contamination and the existence of adventitious carbon on the CNF’s surface [45]. A similar O/C ratio of 0.74 was reported by Paquet et al. [50] for commercial MCC, the type of precursor used in the present study for obtaining the CNF. No variation in the O/C ratio was recorded after the silylation, with a value of 0.75 being obtained for CNF_OTES, although a lower ratio was expected due to the increase in the C atomic concentration in the sample after the grafting with silane moieties containing alkyl chains. This is partially due to the operations conducted during the silylation reaction (especially the final Soxhlet extraction with ethanol) that probably resulted in the removal of the impurities adsorbed on the CNF’s surface [51] and partially due to the low degree of substitution computed from XPS. The removal of adventitious carbon resulted in a decrease in the relative atomic percentage of C from the sample that counteracted the increase expected from the attachment of the OTES grafts.

#### 3.1.3. Static Water Contact Angle

**Figure 4:**
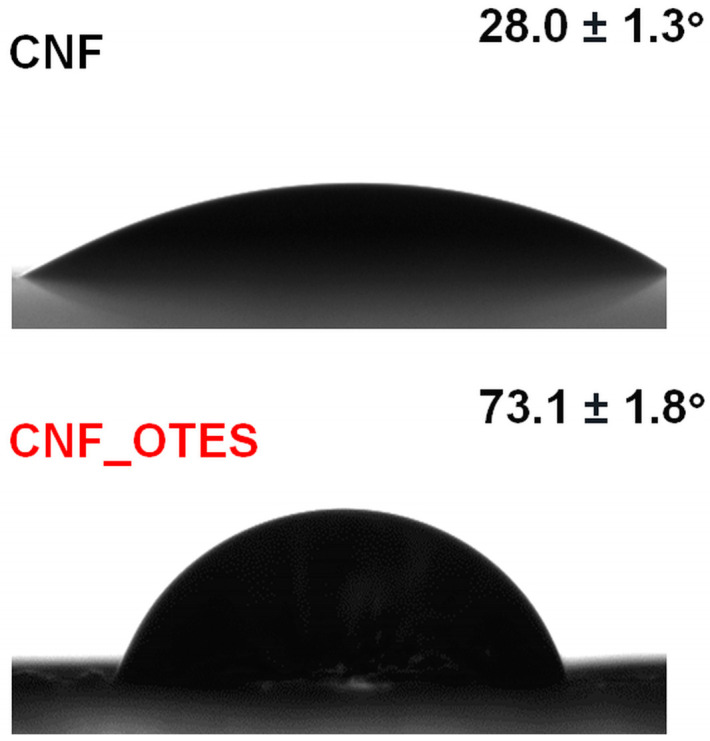
Images of water droplets on the surface of CNF and CNF_OTES during water contact angle measurement.

The changes in the surface hydrophobicity of CNF after the chemical treatment with OTES were investigated by assessing the static water contact angle illustrated in Figure 4. Typically, the higher the hydrophilicity of a material, the lower its contact angle value, while an increase in the water contact angle indicates a rise in the material’s hydrophobicity [52]. Similar to other findings [24,53], unmodified CNF showed a small average contact angle of 28.0 ± 1.3°. This confirms the highly hydrophilic character of CNF, attributed to the abundance of polar OH groups exposed on its surface that are able to bind water and promote CNF’s surface wetting [54].

A more than two-fold increase in the average contact angle to 73.1 ± 1.8° was attained for CNF_OTES, indicating a reduced tendency of the water droplets to wet the CNF’s surface after silylation with OTES [20]. This may constitute another proof that part of the hydrophilic OH groups from the CNFs’ surface was replaced with the hydrophobic moieties of the modifying agent. A similar contact angle of 81° was obtained for bleached eucalyptus pulp fibers grafted with OTES [55] and a higher contact angle of ~110 ± 5° was reported by Jarrah et al. [56] for cellulose cotton fibers grafted with butyltrichloro silane at similar low DS of 0.08 ± 0.03. As previously noted by Mendes et al. [55], silylation with OTES is able to generate a greater increase in the water CA of cellulose as compared to lower-chain-length alkyl silanes, such as isobutyl(trimethoxy)silane and methyltrimethoxy silane, due to the longer aliphatic side group of OTES, but OTES might also have a lower reactivity during the hydrolysis and grafting reactions [19], leading to a lower wetting ability than butyltrichloro silane [56]. Moreover, the presence of oligosiloxane-grafted chains following the self-condensation of OTES, which may shield the hydrophilic OH groups [55] of CNF_OTES, could not be totally ignored.

#### 3.1.4. Thermogravimetric Analysis

**Figure 5:**
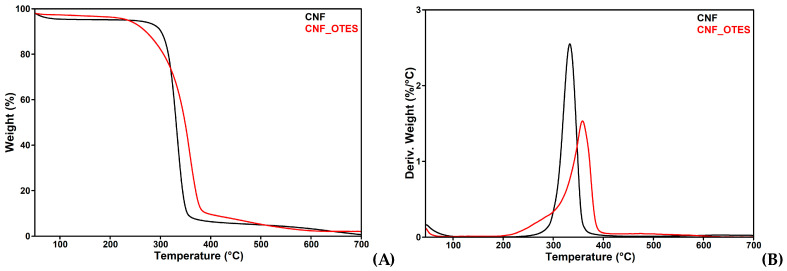
TG (**A**) and DTG (**B**) diagrams of CNF and CNF_OTES.

The TG curves obtained for CNF and CNF_OTES and their first derivative curves (DTG) are shown in Figure 5A,B. CNF begins to lose weight almost immediately after the heating is turned on due to the presence of moisture. A first difference between the treated and untreated CNF samples appears when their weight loss at 100 °C (WL_100 °C_) is compared, CNF_OTES showing much lower WL_100 °C_ than CNF, 2.7% vs. 4.6%, similar to another report [22]. This is an effect of the OTES grafting, which increased the hydrophobicity of CNF and, therefore, the amount of absorbed water. As a consequence of silylation, the temperature at 5% weight loss (T_5%_), the onset degradation temperature (T_onset_), and the temperature at maximum degradation rate (T_max_) are all higher for CNF_OTES than for CNF with 6.3, 6.2, and 25.3 °C (Table 2). This behavior is due to the presence of OTES grafts on the surface of cellulose, which protect it and delay its degradation, being proof of silane grafting on the surface of CNF_OTES. Similar improvement in thermal stability was reported for ground horsetail biomass modified with OTES and other silanes [22], and a smaller increase in thermal stability was reported for CNF grafted with 3-(glycidyloxypropyl) trimethoxy silane [42]. Moreover, the weight loss at 200 °C (WL_200 °C_), a temperature close to that of melt processing of PHB, is lower for CNF_OTES than for CNF, showing an increase in thermal stability after silylation, also proof of the successful grafting.

As expected, the residue at 700 °C (R_700_ °C) is much higher for the silylated nanocellulose than for the untreated sample, 2.1% vs. 0.7% (Table 2). Therefore, in an inert atmosphere under the influence of heat, CNF is mostly pyrolyzed into gases up to 700 °C, while CNF_OTES gives a measurable residual mass related to silicon compounds. This is due to grafted silane because the physically absorbed unreacted silane or oligosiloxanes were removed during the Soxhlet treatment. The higher residue obtained for CHF_OTES is another proof of silane grafting because silicon compounds are generally more stable than organic compounds at 700 °C [57].

#### 3.1.5. Nano-Investigation of CNF and CNF_OTES by AFM-QNM

**Figure 6:**
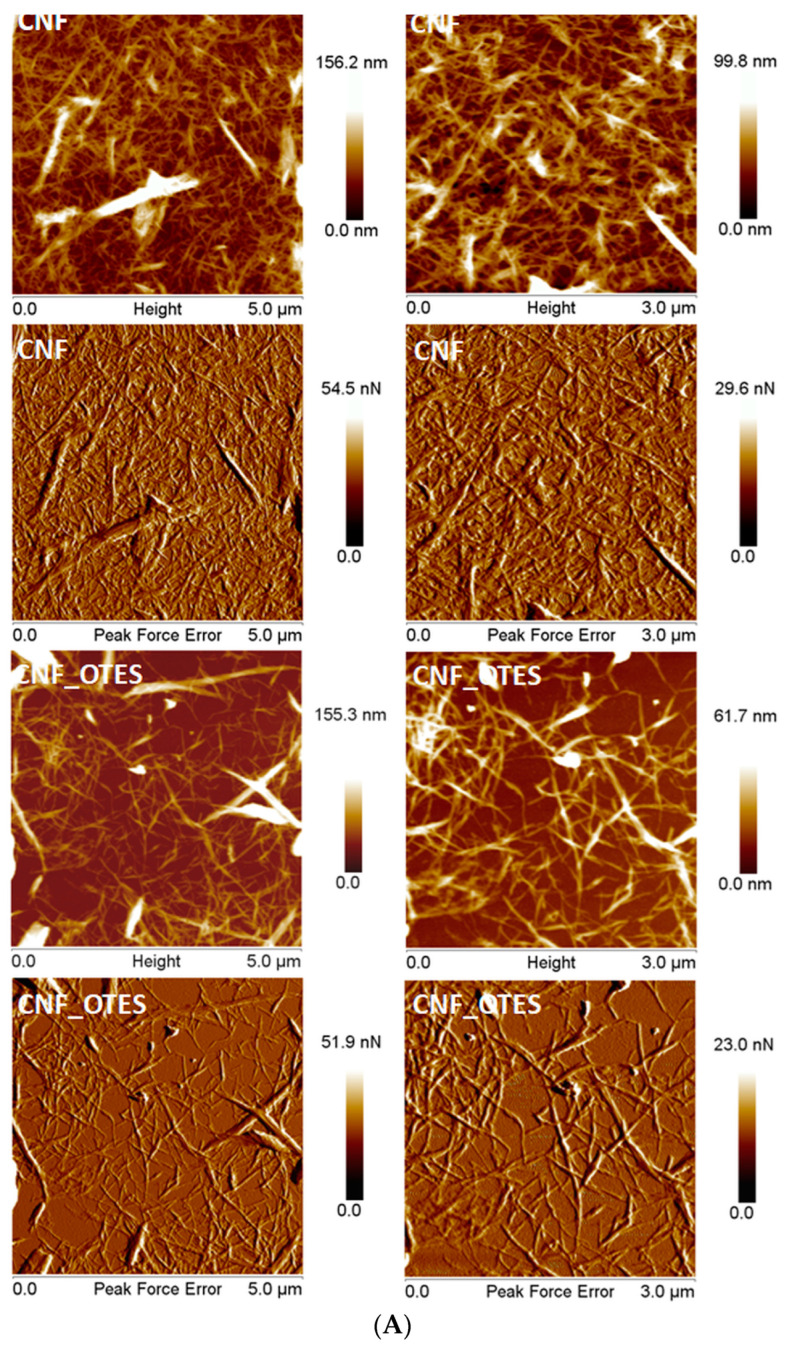

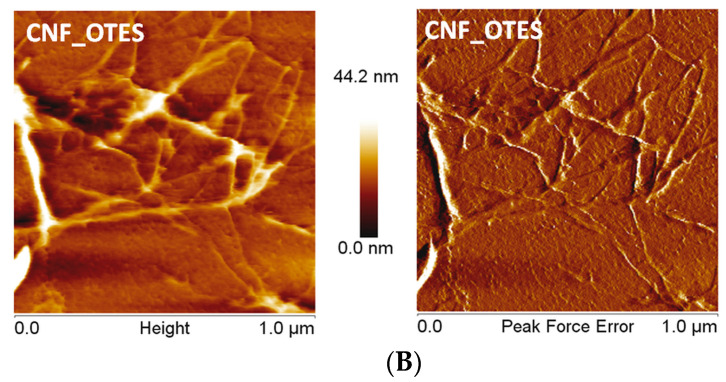
Topographic and PFE images of CNF and CNF_OTES (scanning areas 5 µm × 5 µm and 3 µm × 3 µm) (**A**); topographic and PFE images of CNF_OTES (1 µm× 1 µm) (**B**).

PF QNM mode of AFM is a powerful technique that allows the observation of the morphological features at the nanoscale. A network of individual nanofibers with needle-like shapes was observed in the topographic and PF error (PFE) images of CNF (Figure 6A). The width of these nanofibers varies in a large range, from a couple of nanometers to tens of nanometers. A few fibers or agglomerations of nanofibers with the size of several hundred nanometers could also be observed in the AFM images with a higher scanning area (5 µm × 5 µm) in Figure 6A. The nanofibers’ length varies from hundreds of nanometers to several microns, but their entanglement does not allow the accurate calculation of the length. 

**Figure 7:**
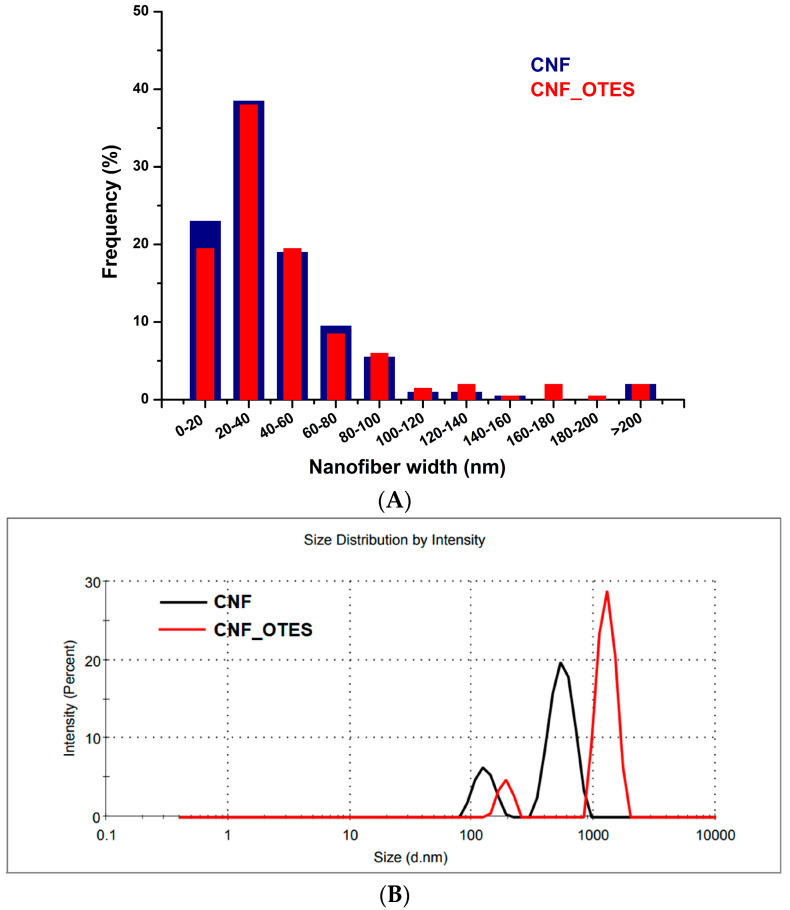
Distribution of cellulose nanofibers’ width resulted from AFM-QNM analysis (**A**); particle size distribution by DLS for CNF and CNF_OTES (**B**).

Although CNF_OTES nanofibers seem to have similar widths and lengths as CNF, their morphological aspect is different, with CNF_OTES nanofibers being much better dispersed and less entangled or agglomerated than CNF. This may be due to the repulsive forces between the alkyl chains grafted on the CNF surface, as reported for polystyrene-grafted silica nanoparticles/polystyrene nanocomposites [58]. An AFM image recorded on a very small CNF_OTES’ surface, of only 1 µm × 1 µm (Figure 6B), shows that well dispersed, fine (7–9 nm) nanofibers were obtained after the treatment of CNF with OTES. A detailed analysis of the nanofibers’ width was carried out with ImageJ (Figure 7A).

Small differences between the widths of CNF and CNF_OTES nanofibers and their size distribution resulted after this analysis. The measurements indicate that the nanofibers’ width is <60 nm for more than 75% of nanofibers in both cases (80.5% for CNF and 77.0% for CNF-OTES), with a very small gain in size for CNF_OTES. This is obvious when the average sizes of nanofibers are compared: 43 ± 14 nm for CNF and 47 ± 18 nm for the silylated nanofibers. The fact that the chemical functionalization of cellulose nanofibers with silane coupling agents only slightly modifies their width was previously reported by Cabrera et al. [42]. Thus, an increase in the average diameter of CNF, from 38 to 52 nm, was noticed after 3-glycidyloxypropyl trimethoxy silane treatment [42]. This tendency was also signaled in the case of other chemical treatments applied to cellulose nanofibers or nanocrystals [59].

#### 3.1.6. DLS Analysis of Water-Dispersed CNF and CNF_OTES

DLS was used to acquire a thorough evaluation of the CNF and CNF_OTES’s sizes when they were dispersed in water. It should be remarked that DLS measures the hydrodynamic radius based on assumptions of spherical particle shapes [60]. Thus, for non-spherical particles, as is the case of CNF and CNF_OTES, which are anisotropic materials with a fibrillar aspect and high aspect ratio, what is determined by DLS is, in fact, the equivalent diameter, i.e., the diameter of a hypothetical sphere with the same diffusion rate as the particle measured [61]. In DLS, the measured hydrodynamic radius of CNFs depends on the lateral dimensions, length, and charge density of the fibers [62]. In fact, DLS is a technique more readily used to estimate the length of cellulose nanostructures [63] rather than their diameter since it has been reported that the diffusion of non-spherical particles—and consequently the DLS-determined average dimensions—are more significantly influenced by changes in length than in diameter [64]. Secondly, in DLS, the measured hydrodynamic radius is strongly dependent on the degree of particle-particle interactions in the dispersion medium that is greatly affected by the concentration of the particles in the system and changes in the particles’ surface chemistry due to modification, which can alter their hydration shell [60]. As it is well known, when suspended in water, cellulose nanofibers rapidly form aggregates of varying sizes [65]. Consequently, the DLS measurements will emphasize particularly the diameters of hypothetical spheres with the same translational diffusion coefficient as the aggregates [66] formed by the random coiling of the CNF and CNF_OTES fibers of different diameters and lengths. Although not suitable for the accurate determination of either the cross-section or length of the CNFs [67,68], DLS might be a useful tool for the comparative study of CNF and CNF-OTES with respect to their overall apparent sizes and aggregation behavior in water [68].

As illustrated in Figure 7B and Table 3, both CNF and CNF_OTES exhibited a bimodal size distribution, with pristine CNF showing a minor peak with a mean diameter at 127.2 nm and a major peak at 553.1 nm, while CNF_OTES having a minor peak with an average diameter at 187.7 nm and a major peak at 1279 nm. This indicates that both the aqueous suspensions of CNF and CNF_OTES contain two distinct size populations: one with finer fibers/aggregates that have smaller diameters and/or lengths and another with larger fibers/aggregates that are longer and/or thicker.

This may allude to the presence of unfibrillated structures [69] in the CNF and CNF_OTES samples but also to their agglomeration in water due to their fine particles and low surface charge [70]. In fact, as stated by Wu et al. [71], fully dispersed cellulose structures scatter little light and become indistinguishable in mixtures where larger ribbons, bundles, and flocs are present. A bimodal distribution of sizes, with the minor peak centered at 105 nm and the major peak at 766 nm, was also attained by Das et al. [72] for CNFs isolated by H_2_SO_4_ hydrolysis of cotton fibers. Similarly, Gamelas et al. [73] reported a bimodal distribution of the dimensions, with one broad peak from about 5 nm to 1000 nm and a second peak around 5000 nm for CNFs produced by TEMPO-oxidation of bleached eucalyptus kraft pulp followed by five passes through a homogenizer.

Nevertheless, a clear increase in the average hydrodynamic diameter occurs in the case of CNF_OTES as compared to pristine CNF. This is likely due to the bulky OTES grafts that may have prevented the CNF_OTES nanofibers from forming as compactly (tightly) coiled aggregates as encountered for pristine CNF, resulting in the formation of larger, more voluminous agglomerates. At the same time, the untreated CNF retained their natural hydrophilicity, allowing them to disperse more effectively in water as individual fibers or smaller bundles. In contrast, functionalization with OTES increased the fibers’ surface hydrophobicity, which potentiated their tendency to agglomerate in water [74].

The ζ-potential measurements revealed that the surface charge characteristics of the cellulose nanofibers have remained virtually unchanged after the chemical modification with the OTES grafts, with a value of −32.08 ± 1.18 mV being recorded for pristine CNF and −31.9 ± 0.84 mV for CNF_OTES. Unmodified cellulose nanofibers present a negative ζ-potential due to the OH groups from their surface that, in water, can dissociate with the release of protons (H^+^) and the formation of a net negative charge on the fibers’ surface [75]. The chemical treatment with OTES left the ζ-potential of CNF unchanged because, unlike other silane agents such as APTES [76], OTES has not in its structure charged groups that can change the surface charge of CNFs. At the same time, since OTES is subjected to hydrolysis prior to grafting onto CNF, n-octylsilanol groups are actually the ones that get attached to CNFs’ surfaces. Similar to the C–OH groups in cellulose, the Si–OH groups from the silanol grafts can get deprotonated and become negatively charged in water [77]. Since the grafting of each silanol moiety comes with the replacement of an OH group in CNF with a silanol moiety containing, depending on the way of binding the silanol grafts, none, one, or two free negatively charged Si–O^−^ groups, the potential is expected to remain negative. Similarly to our findings, Jesionowski et al. [78] reported that the ζ-potential of silica, an inorganic polymer that, similar to cellulose, presents a multitude of OH groups at the surface, did not change after its modification with OTES regardless of the amount of OTES grafted on the silica, due to OTES having a neutral nature. Therefore, OTES grafting will not change the CNF’s surface charge.

### 3.2. PHB/CNF_OTES Nanocomposites

#### 3.2.1. Rheological Characterization

**Figure 8:**
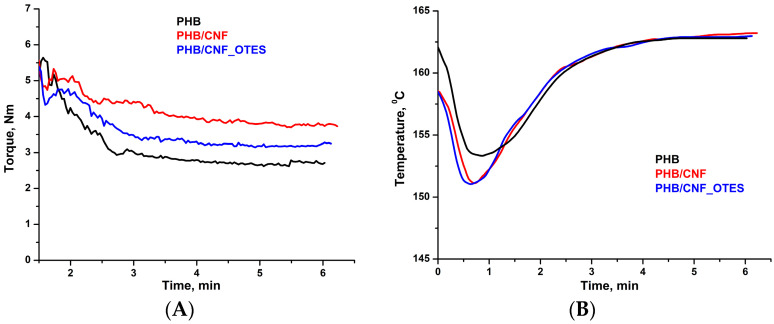
Brabender torque (**A**) and molten material’s temperature (**B**) variation with time for PHB, PHB/CNF, and PHB/CNF_OTES nanocomposites.

Although PHB has many advantageous properties, its melt processability and strength are poor [2]. The addition of nanocellulose in PHB could increase both of these properties. Therefore, the torque vs. time and temperature vs. time curves (Figure 8A,B) of pristine PHB and PHB/CNF and PHB/CNF_OTES nanocomposites were used to analyze the influence of CNF and CNF_OTES on the melting behavior of PHB. The temperature was measured directly in the molten materials with a temperature sensor in permanent contact with the melt. In the first two minutes, the addition of PHB pellets in the Brabender mixer and their melting, followed by the slow addition of nanofibers, led to a noisy variation in the torque values and a decrease in the temperature of the mixing chamber caused by the lower temperature of the added materials and the endothermic melting process [79]. After about 2.5 min, the temperature of the melted materials approaches the set value (160 °C) and continues to increase slightly up to about 163 °C due to the viscous energy dissipation in the PHB matrix [80]. This increase in temperature led to a slight decrease in the melt viscosity, as can be observed from the variation in the torque values with time (Figure 8A).

Although the variation in temperature in the stabilizing region (after about 4 min) is similar for all the samples, the addition of CNF and CNF_OTES in PHB led to an increase in its melt viscosity, the increase being more pronounced in the case of untreated nanofibers (Figure 8A). Higher torque values were previously reported for thermoplastic starch reinforced with CNF as compared with the material without CNF [81] or for PLA/35 *v*/*v*% cellulose microfiber composites as compared to the PLA matrix [79]. This increase may be due to several reasons. In general, the addition of a stiffer filler in a melted polymer increases friction between the solid filler and the polymer matrix and restricts the flow of the polymer melt in the channels between the counter-rotating screws and the walls of the mixing chamber; both of these phenomena lead to an increase in the torque value [82]. However, a further increase in the torque value may be obtained from a stronger adhesion at the filler/polymer interface and also from the formation of filler agglomerates. Both CNF and CNF_OTES are prone to entanglements between their chains and hydrogen bonding between the hydroxyl groups of nanocellulose and the carboxyl groups of PHB due to the low degree of substitution for the silylated nanofibers, which leaves a large percentage of the OH groups suitable for hydrogen bonding in the case of CNF_OTES. However, the presence of a larger proportion of nanofiber agglomerates in the case of CNF than for CNF_OTES can also be presumed based on the AFM results, which showed a greater aggregation tendency in the first case. Additionally, OTES grafts may also act as steric spacers due to their medium-length alkyl chains, which lead to increased flexibility and lower viscosity (lower torque value) [83]. Further characterization of nanocomposites will highlight which of these effects induced by the grafted medium-chain length alkyl silane will prevail.

#### 3.2.2. DMA Results

**Figure 9:**
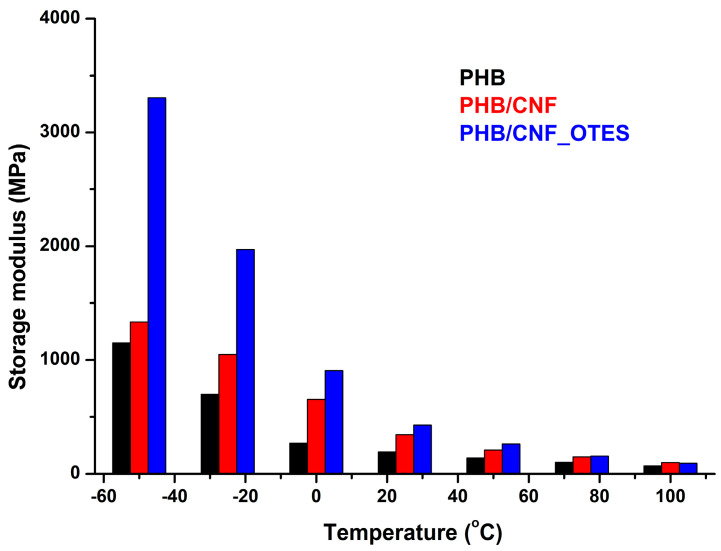
Storage modulus values for PHB and nanocomposites at different temperatures.

The influence of CNF and CNF_OTES on the storage modulus of PHB nanocomposites as compared to the neat PHB is shown in Figure 9. The addition of CNF in PHB increased its storage modulus by 142% at 0 °C and by 78% at 25 °C due to the rigid cellulose nanofibers that introduce constraints to the PHB chains’ mobility, increasing the material stiffness. The addition of CNF_OTES in PHB increased the storage modulus of PHB by 236% at 0 °C and by 122% at 25 °C. The stronger increase in stiffness for the nanocomposite modified with silane-treated cellulose nanofibers is due to higher compatibility between the surface hydrophobized nanofibers and PHB and to an increased interfacial adhesion between the two phases. Indeed, the enhanced interaction at the interface will further restrict the mobility of the PHB chains, leading to much higher stiffness, which was noticed for the nanocomposite with OTES-treated CNF. Similar to our results, an increase in the storage modulus was reported for PHBV reinforced with olive husk flour and a further increase for the composite containing trimethoxyoctadecyl silane-treated flour [16].

The increase in the storage modulus of PHB/CHF and PHB/CHF_OTES, as compared with that of PHB, was less important above room temperature. This was probably determined by the loss of the close packing structure and increased chain mobility with the increase in temperature, which makes the action of the physical interactions at the interface less visible [84].

The different values obtained for the storage modulus of nanocomposites with treated vs. untreated cellulose nanofibers emphasize the different capabilities of these nanocomposites to transfer the stress at the polymer/filler interface [16]. A better interface, which can be presumed for PHB/CHF_OTES, ensures a good distribution and integration of the nanofibers, while a poor one will cause the agglomeration of the nanofibers and more non-uniformities in the material. These aspects related to the dispersion of nanofibers and the occurrence of agglomerates in nanocomposites were examined by SEM.

#### 3.2.3. SEM Analysis

**Figure 10:**
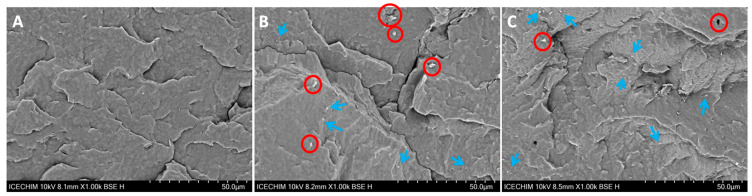
SEM images of the fractured surface of neat PHB (**A**), PHB/CNF (**B**), and PHB/CNF_OTES (**C**) nanocomposites; nanofiber agglomerations were marked with red circles, while well-dispersed nanofibers had blue arrows.

The SEM images of cryo-fractured PHB and nanocomposite samples are shown in Figure 10. The aspect of the fractured samples is characteristic of brittle crystalline materials, which are typical features of PHB in the absence of a plasticizer or an impact modifier [2]. The fractured surface of PHB (Figure 10A) shows a characteristic morphology in the form of terraces, which is due to the presence of crystalline lamellar stacks that guide the fracture direction [85]. The presence of micron-size agglomerations of nanofibers can be more clearly observed at a lower magnification in the SEM images (Figure 10B,C).

Micron-size CNF agglomerations, marked with red circles in Figure 10B, were frequently observed on the fractured surface of PHB/CNF nanocomposite together with well-dispersed nanofibers. This indicates that CNFs form clusters and are unevenly distributed in the section of this nanocomposite. These clusters are weak points from where the cracks can propagate when mechanical stress is applied and explain the lower increase in the storage modulus observed in the case of PHB/CNF nanocomposite [86]. The presence of clusters is extremely rare on the fractured surface of PHB/CHF_OTES, most of the nanofibers being well dispersed and adhering to the PHB matrix. These morphological features may be correlated with the presence of the grafted silane on the surface of CNF_OTES, which ensures good compatibility of nanocellulose with PHB. However, a micron-size hole (marked with a red circle in Figure 10C) was also detected on the fractured surface of PHB/CHF_OTES. This hole may result from pull-out fibers or from broken impurities that are present in the PHB matrix. This rarely observed event in PHB/CNF_OTES may indicate accidental pull-out fibers or poorly integrated CNF_OTES nanofibers in the PHB matrix, leaving areas with small interface adhesion. Contrarily, large cavities located at the filler-matrix interface were frequently observed in the nanocomposite with untreated nanofibers, PHB/CNF, which were marked with red circles in Figure 10B. Actually, the interfacial adhesion ensured by the grafted silane consists of physical interactions through hydrogen bonding due to the presence of OH groups on the surface of CHF_OTES and the ester groups of PHB, along with a multitude of wan der Waals interactions favored by the medium-chain-length alkyl tail of the silane and the hydrophobic chain of PHB, which boost the physical interlocking [87]. These interactions are enough to ensure good dispersion of the CNF_OTS nanofibers in PHB and a remarkable increase in stiffness.

#### 3.2.4. DSC Results

**Figure 11:**
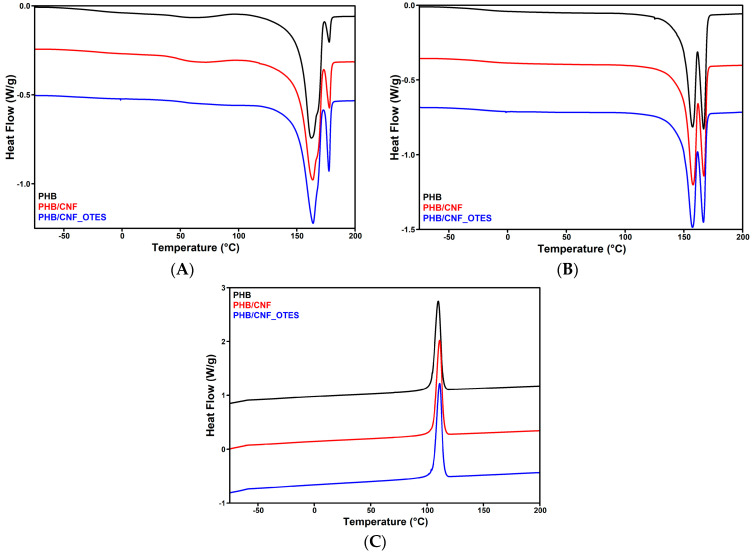
DSC curves of nanocomposites: first heating (**A**), second heating (**B**), and cooling (**C**).

The influence of untreated and silane-treated CNFs on the melting and crystallization behaviors of PHB was analyzed by DSC in non-isothermal conditions. The DSC curves from the first heating, cooling, and second heating cycles are shown in Figure 11A–C, and the most important thermal characteristics are collected in Table 4.

Double melting peaks were observed in the first as well as in the second heating cycles for all the samples, a characteristic behavior of PHB caused probably by the melting–recrystallization–remelting processes or the different perfection of the PHB crystals [14,88,89]. Several differences were observed between the characteristic parameters of the first (I) and second (II) heating processes regarding the melting enthalpies and melting temperatures of the first (1) (ΔH_m1I_, T_m1I_ compared to ΔH_m1II_, T_m1II_) and second (2) (ΔH_m2I_, T_m2I_ compared to ΔH_m2II_, T_m2II_) peaks (Table 4). In the first heating cycle, the first peak is asymmetric and much larger than the second one, while in the second heating cycle, the peaks are closer in size, showing a larger proportion of more perfect crystals [14,88]. The first lower temperature peak is usually ascribed to less perfect, thinner crystals, and the higher temperature peak is related to thicker, more perfect crystals formed during the heating cycle [14,89]. A 5–7 °C decrease in the melting temperature corresponding to the first peak and an 11 °C decrease in the melting temperature of the second peak were noticed for the second heating cycle compared to the first one. This important decrease in the melting temperature after the first heating and cooling cycles illustrates a reduction in the molecular weight of PHB after these thermal cycles due to degradative processes [14,90]. A similar decrease in T_mII_ vs. T_mI_ was reported for PHBV and PHBV reinforced with *Miscanthus* grass fibers [91], for PHBV subjected to aging [92], and for biocomposites from PHB and various bio-fillers [90].

The addition of CNF and CNF_OTES in PHB led to a slight increase in the crystallization temperature, T_m1I_, and crystallinity (Table 4). The slight increase in T_m1I_ and crystallinity could be related to the heterogeneous nucleation effect of CNF and CNF_OTES, which provide new nucleation sites [93]. However, no variation in the other T_m_ values was noticed, which is similar to previous reports [8,94]. Dehouche et al. [94] observed a slight increase in the T_m_ of poly(3-hydroxybutyrate-co-3-hydroxyhexanoate) biocomposites containing unmodified and silane-treated Aloe Vera fibers and no change in the T_m_ for biocomposites containing Aloe Vera fibers subjected to other treatments. Similarly, Chen et al. [8] reported no variation in the T_m_ of PHB composites containing unmodified CNC or CNC modified by ring-opening polymerization of L-lactide. Similar results were reported for PHBV/pistachio shell flour composites [93]. Interestingly, although higher than the crystallinity of PHB, the Xc of PHB/CNF_OTES was not higher than that of the nanocomposite containing untreated CNF. This trend was also observed for PLA nanocomposites with silane-treated CNF vs. untreated CNF [11] or for PHB composites containing pristine or silylated natural fibers [95]. This behavior was explained by the strong bonding between the silylated (nano)fibers and the PLA or PHB matrix, which prevents crystallization to some extent. Thus, it can be supposed that the OTES treatment of CNF ensures a better cellulose nanofiber/PHB interface, which further leads to improved thermal and mechanical properties of the nanocomposites.

#### 3.2.5. TGA Results

**Figure 12:**
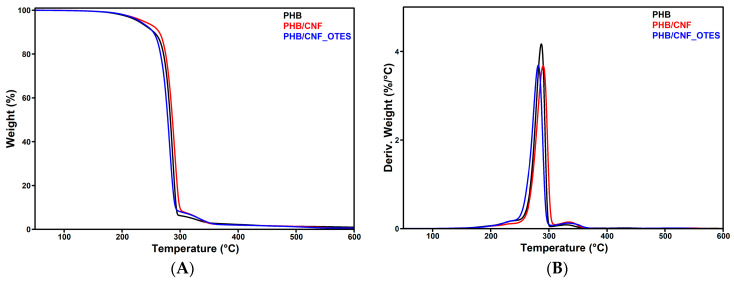
TGA (**A**) and DTG (**B**) curves of PHB nanocomposites.

The changes in the thermal stability of PHB following the addition of CNF and CNF_OTES were studied by TGA. Figure 12 illustrates the (A) thermogravimetric (TG) and (B) differential thermogravimetric (DTG) curves of neat PHB and PHB/CNF and PHB/CNF_OTES nanocomposites. The temperature at 5% weight loss (T_5%_), temperatures at the maximum degradation rates (T_max1_, T_max2_), and the char residue at 700 °C (R_700 °C_) are listed in Table 5.

As shown in Figure 12, neat PHB presents two thermal degradation stages, a main step between 250 and 300 °C and a minor step between 300 and 370 °C. The first thermal degradation stage (250–300 °C) occurs with a drastic reduction in the molecular weight of PHB due to PHB’s depolymerization, which takes place primarily via random chain scission and hydrolysis of the ester bonds. In this phase, PHB mainly degrades via β-elimination at the ester groups, producing crotonic acid and volatile dimeric, trimeric, or tetrametic oligomers of PHB with crotonate end groups. Oligomers larger than tetramers are not sufficiently volatile and, therefore, will remain trapped within the polymer at these temperatures [96]. In the second stage, further decomposition of the PHB oligomers occurs simultaneously with the thermal decomposition of the resulting crotonic acid to propene and carbon dioxide (CO_2_) [97]. In another work, the second thermal degradation stage from the PHB thermograms, occurring between 300 and 400 °C, was attributed to the additives incorporated into the commercial PHB by the manufacturer [98]. It is worth mentioning that another minor degradation stage occurs between 150 and 250 °C for the neat PHB, which can be assigned to the evaporation of free and bound water [91] and/or additives, such as lubricants, found in the commercial PHB used as a polymer matrix [99].

As can be observed from Figure 12B, the PHB/NC and PHB/NC_OTES nanocomposites show the same thermal degradation steps as neat PHB. However, as shown in Table 5, an increase in the T_5%_ by about 9.0 °C and 2.3 °C was observed following the addition of 4 wt% CNF and CNF_OTES to the PHB matrix. This could be due to the CNF nanofillers acting as a physical barrier that slows down the diffusion of heat and volatile degradation products through the PHB matrix and delays the thermal degradation of PHB [100]. Also, the incorporation of CNF and CNF_OTES leaves the thermal stability of PHB generally unchanged, with a 3 °C and 6 °C increase in the T_max1_ and T_max2_ being recorded for PHB/CNF and a 5.3 °C decrease and 5.6 °C increase in the T_max1_ and T_max2_, respectively, being observed for PHB/CNF_OTES. In PHB/CNF, the increase in the T_max1_ and T_max2_ relative to neat PHB could be attributed to the hydrogen bonding interactions between the OH groups of CNF and the C=O groups of the PHB, which decelerated the random chain scission of the PHB matrix, enhancing its thermal stability [3]. The same hydrogen bonding interactions between the OH groups of CNF and the carbonyl groups of the PHB, together with the contributions of the van der Waals interactions between the n-octyl chains of CNF_OTES and the chains of PHB and the hydrogen bonding interactions between the OH groups from the silanol grafts of CNF_OTES and PHB, can explain the small increase in thermal stability of PHB determined by NC_OTES. An increase in the T_max_ of PHB by 4.9–11.7 °C was also reported by Zhang et al. [3] for PHB/CNFs composites containing 1–5 wt% CNFs obtained from bleached cellulose pulp by grinding in an ultrafine pulverizer and defibrillation in a high-pressure homogenizer.

It can be observed in Table 5 that less residue was formed in the case of PHB/CNF and PHB/CNF_OTES as compared to neat PHB. It can be assumed that due to the strong interactions between CNF or CNF_OTES and the PHB matrix, CNF and CNF_OTES interfere with the natural degradation pathway of PHB, altering the formation of stable char and reducing the amount of residue at 700 °C. Similar to our results, a lower amount of char residue was observed for PHBV/cellulose nanowhiskers (CNWs) composites as compared to neat PHBV [101], with strong hydrogen bonding interactions between the CNWs and the PHBV matrix being presumed to explain this behavior. The close R_700 °C_ values obtained for CNF and CNF_OTES are due to the cleavage of the C-Si bond and OTES evaporation at temperatures over 375 °C [102].

Regardless of the case, CNF and CNF_OTES do not significantly affect the thermal stability of PHB, so it can be concluded that the PHB/CNF and PHB/CNF_OTES can be processed under the same conditions as the neat PHB matrix without suffering thermal degradation.

## 4. Conclusions

CNFs were successfully produced from MCC by microfluidization and, for the first time, modified with OTES, a medium-chain-length alkyl silane, using a simple and eco-friendly method. FTIR analysis confirmed the grafting of OTES onto the CNF’s surface, and a degree of silylation of 0.09 was determined based on the XPS survey results. Treatment with OTES led to a rise in the thermal stability of CNF, reflected in an increase in the T_max_ from 333.1 to 358.4 °C. AFM analysis revealed that the nanofibers maintained their nano diameters after the silylation reaction, while the substantial increase in the water CA from 28 ± 1.3° to 73.1 ± 1.8° indicated an increase in the CNF’s surface hydrophobicity following the chemical treatment with OTES, making CNF-OTES a promising reinforcing agent for a hydrophobic PHB matrix. The PHB/CNF-OTES composites containing 4 wt% silylated CNF exhibited a 122% increase in the storage modulus at 25 °C as compared to the neat PHB, which is significantly greater than the improvement determined by the untreated CNFs (78%), suggesting that chemical modification with OTES enhanced the compatibility between the CNF and the PHB matrix, leading to an improved PHB/fiber interface. The DMA results were supported by the SEM images, which revealed a good overall dispersion of the CNF_OTES nanofibers in the PHB matrix. The DSC results indicated that both CNF and CNF_OTES acted as nucleating agents for PHB, leading to a slight increase in the Xc of PHB from 52.2% to 55.1 and 54.6%, respectively, while the TGA demonstrated that the reinforcement with CNF_OTES did not alter the thermal stability of PHB. All these results recommend CNF_OTES as a suitable nanofiller for PHB or other hydrophobic biopolymers; this is an additional step towards obtaining polymeric nanocomposites that are entirely bio-based, biodegradable, and biocompatible, with more potential applications across various fields. Further characterization of these nanocomposites through specific tests such as gas barrier, water absorption, migration, optical properties, and toxicity will better define the feasibility of these materials for targeted applications such as food packaging.

## Figures and Tables

**Table 1:** The surface elemental composition and C/O atomic ratio for CNF and CNF_OTES as determined from the XPS survey spectra.

Sample	O1s (at%)	C1s (at%)	Si2p (at%)	O/C
CNF	42.61	57.39	-	0.74
CNF_OTES	42.65	56.65	0.7	0.75

**Table 2:** Thermogravimetric characterization results for CNF and CNF_OTES.

Samples	WL_100 °C_ (%)	T_5%_ (°C)	T_onset_ (°C)	T_max_ (°C)	WL_200 °C_ (%)	R_700 °C_ (%)
CNF	4.6	227.9	314.1	333.1	4.9	0.7
CNF_OTES	2.7	234.2	320.3	358.4	3.7	2.1

**Table 3:** The average diameters corresponding to the 1st peak (D_m1_) and the 2nd peak (D_m2_), polydispersity index (PdI), and ζ-potential for CNF and CNF_OTES.

Sample	D_m1_ (nm)	D_m2_(nm)	PdI	ζ-Potential (mV)
CNF	140.7 ± 14.2	593.2 ± 49.7	0.82 ± 0.11	−32.08 ± 1.18
CNF_OTES	150.2 ± 25.9	1267.8 ± 144.9	0.73 ± 0.09	−31.90 ± 0.84

**Table 4:** Melting and crystallization characteristics for PHB and nanocomposites.

Samples	First Heating				Cooling		Second Heating				Xc, %
	T_m1I_, °C	ΔH_m1I_, J/g	T_m2I_, °C	ΔH_m2I_, J/g	T_c_,°C	ΔH_c_, J/g	T_m1II_, °C	ΔH_m1II_, J/g	T_m2II_, °C	ΔH_m2II_, J/g	
PHB	162.4	71.9	177.7	3.0	109.8	66.7	157.5	51.1	166.8	25.1	52.2
PHB/CNF	163.5	69.2	177.9	6.1	111.0	66.9	157.7	53.1	166.9	23.3	54.5
PHB/CNF_OTES	163.9	65.3	177.5	8.2	111.0	66.3	157.4	52.7	166.5	23.1	54.1

**Table 5:** Thermogravimetric characteristics for PHB nanocomposites.

Samples	T_5%_ (°C)	T_max1_(°C)	T_max2_(°C)	R_700 °C_ (%)
PHB	227.9	287.1	330.9	0.87
PHB/CNF	236.9	290.1	336.9	0.56
PHB/CNF_OTES	230.2	281.8	336.5	0.56

## Data Availability

Data are contained within the article.

## References

[1] Muthuraj R., Valerio O., Mekonnen T.H. (2021). Recent developments in short- and medium-chain- length Polyhydroxyalkanoates: Production, properties, and applications. Int. J. Biol. Macromol..

[2] Popa M.S., Frone A.N., Panaitescu D.M. (2022). Polyhydroxybutyrate blends: A solution for biodegradable packaging?. Int. J. Biol. Macromol..

[3] Zhang B., Huang C., Zhao H., Wang J., Yin C., Zhang L., Zhao Y. (2019). Effects of Cellulose Nanocrystals and Cellulose Nanofibers on the Structure and Properties of Polyhydroxybutyrate Nanocomposites. Polymers.

[4] Usurelu C.D., Badila S., Frone A.N., Panaitescu D.M. (2022). Poly(3-hydroxybutyrate) Nanocomposites with Cellulose Nanocrystals. Polymers.

[5] Noremylia M.B., Hassan M.Z., Ismail Z. (2022). Recent advancement in isolation, processing, characterization and applications of emerging nanocellulose: A review. Int. J. Biol. Macromol..

[6] Panaitescu D.M., Frone A.N. (2023). Cellulose (Nano) Composites. Polymers.

[7] Yu H.Y., Qin Z.Y., Yan C.F., Yao J.M. (2014). Green nanocomposites based on functionalized cellulose nanocrystals: A study on the relationship between interfacial interaction and property enhancement. ACS Sustain. Chem. Eng..

[8] Chen J., Wu D., Tam K.C., Pan K., Zheng Z. (2017). Effect of surface modification of cellulose nanocrystal on nonisothermal crystallization of poly(β-hydroxybutyrate) composites. Carbohydr. Polym..

[9] Magnani C., Idström A., Nordstierna L., Müller A.J., Dubois P., Raquez J.M., Lo Re G. (2020). Interphase design of cellulose nanocrystals/poly(hydroxybutyrate-ran-valerate) bionanocomposites for mechanical and thermal properties tuning. Biomacromolecules.

[10] Zhou L., Ke K., Yang M.-B., Yang W. (2021). Recent progress on chemical modification of cellulose for high mechanical-performance Poly(lactic acid)/Cellulose composite: A review. Compos. Commun..

[11] Frone A.N., Berlioz S., Chailan J.-F., Panaitescu D.M. (2013). Morphology and thermal properties of PLA–cellulose nanofibers composites. Carbohydr. Polym..

[12] Popa M.S., Frone A.N., Radu I.C., Stanescu P.O., Truşcă R., Rădiţoiu V., Nicolae C.A., Gabor A.R., Panaitescu D.M. (2021). Microfibrillated Cellulose Grafted with Metacrylic Acid as a Modifier in Poly(3-hydroxybutyrate). Polymers.

[13] Panaitescu D.M., Vizireanu S., Stoian S.A., Nicolae C.-A., Gabor A.R., Damian C.M., Trusca R., Carpen L.G., Dinescu G. (2020). Poly(3-hydroxybutyrate) Modified by Plasma and TEMPO-Oxidized Celluloses. Polymers.

[14] Wang B., Sain M. (2007). The effect of chemically coated nanofiber reinforcement on biopolymer based nanocomposites. BioResources.

[15] Panaitescu D.M., Nicolae C.A., Gabor A.R., Trusca R. (2020). Thermal and mechanical properties of poly(3-hydroxybutyrate) reinforced with cellulose fibers from wood waste. Ind. Crops Prod..

[16] Hassaini L., Kaci M., Touati N., Pillin I., Kervoelen A., Bruzaud S. (2017). Valorization of olive husk flour as a filler for biocomposites based on poly(3-hydroxybutyrate-co-3-hydroxyvalerate): Effects of silane treatment. Polym. Test..

[17] Ou J., Wang F., Li W., Yan M., Amirfazli A. (2020). Methyltrimethoxysilane as a multipurpose chemical for durable superhydrophobic cotton fabric. Prog. Org. Coat..

[18] Giubilini A., Sciancalepore C., Messori M., Bondioli F. (2020). New biocomposite obtained using poly(3-hydroxybutyrate-co-3-hydroxyhexanoate) (PHBH) and microfibrillated cellulose. J. Appl. Polym. Sci..

[19] Pyo C.E., Chan J.H. (2021). Hydrophobic mesoporous silica particles modified with nonfluorinated alkyl silanes. ACS Omega.

[20] El Allaoui B., Chakhtouna H., Zari N., Benzeid H., Qaiss A.e.k., Bouhfid R. (2023). Superhydrophobic alkylsilane functionalized cellulose beads for efficient oil/water separation. J. Water Process Eng..

[21] Taczała J., Sawicki J., Pietrasik J. (2020). Chemical Modification of Cellulose Microfibres to Reinforce Poly(methyl methacrylate) Used for Dental Application. Materials.

[22] Miedzianowska J., Masłowski M., Strzelec K. (2023). Improving performance of natural rubber composites by the application of functional biofiller: Horsetail modified with silane coupling agents. Cellulose.

[23] Oprea M., Panaitescu D.M., Nicolae C.A., Gabor A.R., Frone A.N., Raditoiu V., Trusca R., Casarica A. (2020). Nanocomposites from functionalized bacterial cellulose and poly(3-hydroxybutyrate-co-3-hydroxyvalerate. Polym. Degrad. Stab..

[24] Andresen M., Johansson L.S., Tanem B.S., Stenius P. (2006). Properties and characterization of hydrophobized microfibrillated cellulose. Cellulose.

[25] Frone A.N., Panaitescu D.M., Chiulan I., Nicolae C.A., Casarica A., Gabor A.R., Trusca R., Damian C.M., Purcar V., Alexandrescu E. (2018). Surface Treatment of Bacterial Cellulose in Mild, Eco-Friendly Conditions. Coatings.

[26] Jančič U., Bračič M., Ojstršek A., Božič M., Mohan T., Gorgieva S. (2021). Consolidation of cellulose nanofibrils with lignosulphonate bio-waste into excellent flame retardant and UV blocking membranes. Carbohydr. Polym..

[27] Paniz O.G., Pereira C.M.P., Pacheco B.S., Wolke S.I., Maron G.K., Mansilla A., Colepicolo P., Orlandi M.O., Osorio A.G., Carreño N.L.V. (2020). Cellulosic material obtained from antarctic algae biomass. Cellulose.

[28] Alamri H., Low I.M. (2012). Mechanical properties and water absorption behaviour of recycled cellulose fibre reinforced epoxy composites. Polym. Test..

[29] Sekine Y., Nankawa T., Hiroi K., Oba Y., Nagakawa Y., Sugita T., Shibayama Y., Ikeda-Fukazawa T. (2024). Nanocellulose hydrogels formed via crystalline transformation from cellulose I to II and subsequent freeze cross-linking reaction. Carbohydr. Polym..

[30] Rawat S., Misra N., Shelkar S.A., Kumar V. (2023). Tailoring acid free-paper based analytical devices (Af-PADs) via radiation assisted modification of cellulose paper. Carbohydr. Polym..

[31] Kotov N., Larsson P.A., Jain K., Abitbol T., Cernescu A., Wågberg L., Johnson C.M. (2023). Elucidating the fine-scale structural morphology of nanocellulose by nano infrared spectroscopy. Carbohydr. Polym..

[32] Chen Z., Hu T.Q., Jang H.F., Grant E. (2015). Modification of xylan in alkaline treated bleached hardwood kraft pulps as classified by attenuated total-internal-reflection (ATR) FTIR Spectroscopy. Carbohydr. Polym..

[33] Liu Y., Liu A., Ibrahim S.A., Yang H., Huang W. (2018). Isolation and characterization of microcrystalline cellulose from pomelo peel. Int. J. Biol. Macromol..

[34] Arun V., Perumal E.M., Prakash K.A., Rajesh M., Tamilarasan K. (2020). Sequential fractionation and characterization of lignin and cellulose fiber from waste rice bran. J. Environ. Chem. Eng..

[35] Refaat A., Elhaes H., Ibrahim M.A. (2023). Effect of alkali metals on physical and spectroscopic properties of cellulose. Sci. Rep..

[36] Costa G.R., Nascimento M.V., de Souza Marotti B., Arantes V. (2024). Reducing hydrophilicity of cellulose nanofibrils through lipase-catalyzed surface engineering with renewable grafting agents. J. Polym. Environ..

[37] Carrillo I., Mendonça R.T., Ago M., Rojas O.J. (2018). Comparative study of cellulosic components isolated from different eucalyptus species. Cellulose.

[38] Lee H., Erwin A., Buxton M.L., Kim M., Stryutsky A.V., Shevchenko V.V., Sokolov A.P., Tsukruk V.V. (2021). Shape persistent, highly conductive ionogels from ionic liquids reinforced with cellulose nanocrystal network. Adv. Funct. Mater..

[39] Akbarian-Saravi N., Basar I.A., Margoto O.H., Abdollahi G.N., Crawford B., Magel B., Gharibnavaz M., Eskicioglu C., Milani A.S. (2024). Characterization of the mechanical, biodegradation, and morphological properties of nbr/biopolymer blend, integrated with a risk evaluation. ACS Omega.

[40] Sivaranjini B., Mangaiyarkarasi R., Ganesh V., Umadevi S. (2018). Vertical alignment of liquid crystals over a functionalized flexible substrate. Sci. Rep..

[41] Petcu C., Purcar V., Ianchiş R., Spătaru C.I., Ghiurea M., Nicolae C.A., Stroescu H., Atanase L.I., Frone A.N., Trică B. (2016). Synthesis and characterization of polymer-silica hybrid latexes and sol-gel-derived films. Appl. Surf. Sci..

[42] Cabrera I.C., Berlioz S., Fahs A., Louarn G., Carriere P. (2020). Chemical functionalization of nano fibrillated cellulose by glycidyl silane coupling agents: A grafted silane network characterization study. Int. J. Biol. Macromol..

[43] Zhu Q., Wang T., Sun X., Wei Y., Zhang S., Wang X., Luo L. (2022). Effects of Fluorine-Based Modification on Triboelectric Properties of Cellulose. Polymers.

[44] Kaynak B., Spoerk M., Shirole A., Ziegler W., Sapkota J., Kaynak B., Ziegler W., Spoerk M., Sapkota J., Shirole A. (2018). Polypropylene/cellulose composites for material extrusion additive manufacturing. Macromol. Mater. Eng..

[45] Dhali K., Daver F., Cass P., Adhikari B. (2022). Surface modification of the cellulose nanocrystals through vinyl silane grafting. Int. J. Biol. Macromol..

[46] Chen S., Teng Q. (2017). Quantitative Immobilization of Phthalocyanine onto Bacterial Cellulose for Construction of a High-Performance Catalytic Membrane Reactor. Materials.

[47] Chen Q., Xiong J., Chen G., Tan T. (2020). Preparation and characterization of highly transparent hydrophobic nanocellulose film using corn husks as main material. Int. J. Biol. Macromol..

[48] Coelho Braga de Carvalho A.L., Ludovici F., Goldmann D., Silva A.C., Liimatainen H. (2021). Silylated thiol-containing cellulose nanofibers as a bio-based flocculation agent for ultrafine mineral particles of chalcopyrite and pyrite. J. Sustain. Met..

[49] Xu C.A., Lu M., Wu K., Shi J. (2021). Functionalization of nano-cellulose by coupling agent with green strategy. Inorg. Chem. Commun..

[50] Paquet O., Krouit M., Bras J., Thielemans W., Belgacem M.N. (2010). Surface Modification of Cellulose by PCL Grafts. Acta Mater..

[51] Lalanne-Tisné M., Mees M.A., Eyley S., Zinck P., Thielemans W. (2020). Organocatalyzed ring opening polymerization of lactide from the surface of cellulose nanofibrils. Carbohydr. Polym..

[52] Song H., Zheng L. (2013). Nanocomposite films based on cellulose reinforced with nano-SiO_2_: Microstructure, hydrophilicity, thermal stability, and mechanical properties. Cellulose.

[53] Qing Y., Cai Z., Wu Y., Yao C., Wu Q., Li X. (2015). Facile Preparation of optically transparent and hydrophobic cellulose nanofibril composite films. Ind. Crops Prod..

[54] Nazri A.I., Ahmad A.L., Hussin M.H. (2021). Microcrystalline Cellulose-Blended Polyethersulfone Membranes for Enhanced Water Permeability and Humic Acid Removal. Membranes.

[55] Mendes R.F., Mendes L.M., Elvis De Oliveira J., Junior H.S., Glenn G., Henrique G., Tonoli D. (2015). Modification of Eucalyptus Pulp Fiber Using Silane Coupling Agents With Aliphatic Side Chains of Different Length. Polym. Eng. Sci..

[56] Jarrah K., Hisaindee S., Al-Sayah M.H. (2018). Preparation of Oil Sorbents by Solvent-Free Grafting of Cellulose Cotton Fibers. Cellulose.

[57] Sehlleier Y.H., Abdali A., Schnurre S.M., Wiggers H., Schulz C. (2014). Surface functionalization of microwave plasma-synthesized silica nanoparticles for enhancing the stability of dispersions. J. Nanopart. Res..

[58] Chevigny C., Dalmas F., Di Cola E., Gigmes D., Bertin D., Boué F., Jestin J. (2011). Polymer-grafted-nanoparticles nanocomposites: Dispersion, grafted chain conformation, and rheological behavior. Macromolecules.

[59] Miri N.E., Heggset E.B., Wallsten S., Svedberg A., Syverud K., Norgren M. (2022). A comprehensive investigation on modified cellulose nanocrystals and their films properties. Int. J. Biol. Macromol..

[60] Qua E.H., Hornsby P.R., Sharma H.S.S., Lyons G. (2011). Preparation and characterisation of cellulose nanofibres. J. Mater. Sci..

[61] Yang H., Alam M.N., van de Ven T.G.M. (2013). Highly charged nanocrystalline cellulose and dicarboxylated cellulose from periodate and chlorite oxidized cellulose fibers. Cellulose.

[62] Moser C., Lindström M.E., Henriksson G. (2015). Toward industrially feasible methods for following the process of manufacturing cellulose nanofibers. Bioresources.

[63] Tarrés Q., Aguado R., Zoppe J.O., Mutjé P., Fiol N., Delgado-Aguilar M. (2022). Dynamic Light Scattering Plus Scanning Electron Microscopy: Usefulness and Limitations of a Simplified Estimation of Nanocellulose Dimensions. Nanomaterials.

[64] Gorgieva S., Vogrinčič R., Kokol V. (2015). Polydispersity and assembling phenomena of native and reactive dye-labelled nanocellulose. Cellulose.

[65] Das M., Bhattacharyya R. (2015). Cellulose Nanofibers: Synthesis, Properties and Applications. Polymer Nanocomposites Based on Inorganic and Organic Nanomaterials.

[66] Jiang F., Kondo T., Hsieh Y.L. (2016). Rice straw cellulose nanofibrils via aqueous counter collision and differential centrifugation and their self-assembled structures. ACS Sustain. Chem. Eng..

[67] Jakubek Z.J., Chen M., Couillard M., Leng T., Liu L., Zou S., Baxa U., Clogston J.D., Hamad W.Y., Johnston L.J. (2018). Characterization challenges for a cellulose nanocrystal reference material: Dispersion and particle size distributions. J. Nanopart. Res..

[68] Nagarajan K.J., Ramanujam N.R., Sanjay M.R., Siengchin S., Surya Rajan B., Sathick Basha K., Madhu P., Raghav G.R. (2021). A comprehensive review on cellulose nanocrystals and cellulose nanofibers: Pretreatment, preparation, and characterization. Polym. Compos..

[69] Yamagata Y., Niinobe S., Suga K., Nakano Y., Miyamoto K. (2022). Rheological and rheo-optical behaviors of nanocellulose suspensions containing unfibrillated fibers. Cellulose.

[70] Cebreiros F., Seiler S., Sánchez G., Lareo C. (2024). Sequential ball milling as a promising method for the isolation of cellulose nanofibers (CNF) from enzyme-treated eucalyptus kraft pulp. Ind. Crops Prod..

[71] Wu X., Babi M., Moran-Mirabal J., Pelton R.H. (2024). Grafting Polyanhydride Polymers to Cellulose Nanofibers. Cellulose.

[72] Das D., Bhattacharjee S., Bhaladhare S. (2023). Preparation of cellulose hydrogels and hydrogel nanocomposites reinforced by crystalline cellulose nanofibers (CNFs) as a water reservoir for agriculture use. ACS Appl. Polym. Mater..

[73] Gamelas J.A.F., Pedrosa J., Lourenço A.F., Mutjé P., González I., Chinga-Carrasco G., Singh G., Ferreira P.J.T. (2015). On the morphology of cellulose nanofibrils obtained by TEMPO-mediated oxidation and mechanical treatment. Micron.

[74] Neves R.M., Ornaghi H.L., Zattera A.J., Amico S.C. (2020). The influence of silane surface modification on microcrystalline cellulose characteristics. Carbohydr. Polym..

[75] Hongrattanavichit I., Aht-Ong D. (2021). Antibacterial and water-repellent cotton fabric coated with organosilane-modified cellulose nanofibers. Ind. Crops Prod..

[76] Najib N., Christodoulatos C. (2019). Removal of arsenic using functionalized cellulose nanofibrils from aqueous solutions. J. Hazard. Mater..

[77] Chen Z., Hsu F.C., Battigelli D., Chang H.C. (2006). Capture and release of viruses using amino-functionalized silica particles. Anal. Chim. Acta.

[78] Jesionowski T., Ciesielczyk F., Krysztafkiewicz A. (2010). Influence of selected alkoxysilanes on dispersive properties and surface chemistry of spherical silica precipitated in emulsion media. Mater. Chem. Phys..

[79] Patti A., Acierno D., Latteri A., Tosto C., Pergolizzi E., Recca G., Cristaudo M., Cicala G. (2020). Influence of the Processing Conditions on the Mechanical Performance of Sustainable Bio-Based PLA Compounds. Polymers.

[80] Teuber L., Militz H., Krause A. (2016). Processing of wood plastic composites: The influence of feeding method and polymer melt flow rate on particle degradation. J. Appl. Polym. Sci..

[81] Son D., Lee J., Kim S.K., Hong J., Jung H., Shim J.K., Kang D.H. (2024). Effect of cellulose nanofiber-montmorillonite hybrid filler on the melt blending of thermoplastic starch composites. Int. J. Biol. Macromol..

[82] Koay S.C., Chan M.Y., Pang M.M., Tshai K.Y. (2018). Influence of filler loading and palm oil-based green coupling agent on torque rheological properties of polypropylene/cocoa pod husk composites. Adv. Polym. Technol..

[83] Romo-Uribe A. (2001). On the molecular orientation and viscoelastic behaviour of liquid crystalline polymers: The influence of macromolecular architecture. Proc. R. Soc. Lond. A.

[84] Nandi P., Das D. (2023). Mechanical, thermo-mechanical and biodegradation behaviour of surface-silanized nettle fabric-reinforced poly (lactic acid) composites. Mater. Chem. Phys..

[85] Panaitescu D.M., Trusca R., Gabor A.R., Nicolae C.A., Casarica A. (2020). Biocomposite foams based on polyhydroxyalkanoate and nanocellulose: Morphological and thermo-mechanical characterization. Int. J. Biol. Macromol..

[86] de Oliveira P.F., de Oliveira Aguiar V., Marques M.F.V., Monteiro S.N. (2024). Effect of acid treatment of eucalyptus fibers for improved poly(3-hydroxybutyrate) nanocomposites. J. Mater. Res. Technol..

[87] Song W., Yang Z., Zhang S., Fei B., Zhao R. (2023). Properties enhancement of poly(β-hydroxybutyrate) biocomposites by incorporating surface-modified wheat straw flour: Effect of pretreatment methods. Int. J. Biol. Macromol..

[88] Wellen R.M.R., Rabello M.S., Fechine G.J.M., Canedo E.L. (2013). The melting behaviour of poly(3-hydroxybutyrate) by DSC. Reproducibility study. Polym. Test..

[89] Laycock B., Halley P., Pratt S., Werker A., Lant P. (2013). The chemomechanical properties of microbial polyhydroxyalkanoates. Prog. Polym. Sci..

[90] Meereboer K.W., Pal A.K., Cisneros-López E.O., Misra M., Mohanty A.K. (2021). The effect of natural fillers on the marine biodegradation behaviour of poly(3-hydroxybutyrate-co-3-hydroxyvalerate) (PHBV). Sci. Rep..

[91] Uzun G., Aydemir D. (2017). Biocomposites from polyhydroxybutyrate and bio-fillers by solvent casting method. Bull. Mater. Sci..

[92] Deroiné M., Le Duigou A., Corre Y.-M., Le Gac P.-Y., Davies P., César G., Bruzaud S. (2014). Seawater accelerated ageing of poly(3-hydroxybutyrate-co-3-hydroxyvalerate). Polym. Degrad. Stab..

[93] Altun M., Çelebi M., Şen S., Kökpınar Ö., Kaçoğlu H.S. (2023). Characterization of polyhydroxyalkanoate-based composites derived from waste cooking oil and agricultural surplus. Polym. Compos..

[94] Dehouche N., Idres C., Kaci M., Zembouai I., Bruzaud S. (2020). Effects of various surface treatments on Aloe Vera fibers used as reinforcement in poly(3-hydroxybutyrate-co-3-hydroxyhexanoate) (PHBHHx) biocomposites. Polym. Degrad. Stab..

[95] Mlhem A., Abu-Jdayil B., Iqbal M.Z. (2023). High-performance, renewable thermal insulators based on silylated date palm fiber–reinforced poly(β-hydroxybutyrate) composites. Dev. Built Environ..

[96] Gonzalez A., Irusta L., Fernández-Berridi M.J., Iriarte M., Iruin J.J. (2005). Application of pyrolysis/gas chromatography/Fourier transform infrared spectroscopy and TGA techniques in the study of thermal degradation of poly(3-hydroxybutyrate). Polym. Degrad. Stab..

[97] Clark J.M., Pilath H.M., Mittal A., Michener W.E., Robichaud D.J., Johnson D.K. (2016). Direct production of propene from the thermolysis of poly(β-hydroxybutyrate) (PHB). An experimental and DFT investigation. J. Phys. Chem. A.

[98] D’Arienzo L., Acierno S., Patti A., Di Maio L. (2024). Cellulose/Polyhydroxybutyrate (PHB) Composites as a Sustainable Bio-Based Feedstock to 3D-Printing Applications. Materials.

[99] Arrieta M.P., Samper M.D., Aldas M., López J. (2017). On the Use of PLA-PHB Blends for Sustainable Food Packaging Applications. Materials.

[100] Mahmood H., Pegoretti A., Brusa R.S., Ceccato R., Penasa L., Tarter S., Checchetto R. (2020). Molecular transport through 3-hydroxybutyrate co-3-hydroxyhexanoate biopolymer films with dispersed graphene oxide nanoparticles: Gas barrier, structural and mechanical properties. Polym. Test..

[101] Martínez-Sanz M., Vicente A.A., Gontard N., Lopez-Rubio A., Lagaron J.M. (2015). On the extraction of cellulose nanowhiskers from food by-products and their comparative reinforcing effect on a polyhydroxybutyrate-co-valerate polymer. Cellulose.

[102] López-Aranguren P., Saurina J., Vega L.F., Domingo C. (2012). Sorption of tryalkoxysilane in low-cost porous silicates using a supercritical CO_2_ method. Microporous Mesoporous Mater..

